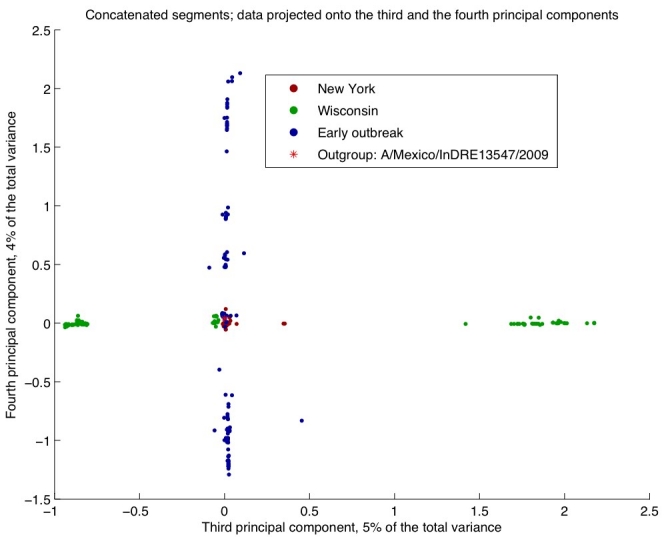# Host Dependent Evolutionary Patterns and the Origin of 2009 H1N1 Pandemic Influenza

**DOI:** 10.1371/currents.RRN1147

**Published:** 2010-08-04

**Authors:** Alexander Solovyov, Benjamin Greenbaum, Gustavo Palacios, W. Ian Lipkin, Raul Rabadan

**Affiliations:** ^*^Columbia Unoiversity; ^†^Simons Center for Systems Biology, Institute for Advanced Study; ^‡^Center for Infection and Immunity and ^§^Columbia University

## Abstract

The origin of H1N1pdm constitutes an unresolved mystery, as its most recently observed ancestors were isolated in pigs nearly a decade before it emerged in humans. One theory suggests imperfect surveillance of swine viruses caused the virus to be missed in swine herds. Other hypotheses point to the possibility of laboratory error or an avian intermediary. We show substitution bias classification identifies the host where a virus has been evolving. Comparing the evolution of H1N1pdm ancestors with other influenza viruses, we show the evolutionary history in unsampled years is similar to the evolution of other swine viruses, presenting evidence it emerged from unsampled herds.

## Introduction

The recent spread of 2009 H1N1 pandemic influenza has shown the need to identify emerging pathogens as quickly as possible.  In less than two months after it was first reported in March 2009, the virus had been detected in all habitable continents.  Influenza is a segmented single-stranded RNA virus, and like many other RNA viruses, it has high mutational and evolutionary rates, more than 5 orders of magnitude greater than that of Human DNA [1]. The genomic similarity of different H1N1 pandemic isolates suggests a recent common ancestor, probably from the beginning of 2009, followed by local geographic spreading [2]. This virus was formed by a reassortment of two swine viruses [3] [4] [5], one of them contributing the M and NA segments. The closest isolates to the strain that contributed the other six segments (including the three polymerases that constitute half of its genome) were found near the turn of the century. They were related to triple reassortant viruses isolated in North America since 1998 [3].

It is interesting to note that none of the direct ancestors of the H1N1 pandemic virus were seen for almost a decade. The ‘unsampled pig herd’ hypothesis suggests that, due to the poor sampling of swine influenza A viruses in several parts of the world, the ancestors of the pandemic virus could have well passed unnoticed [3] [4] [5] [6]. Other hypotheses, such as the one presented by Gibbs et al [7], suggest a non-natural process, like an accident in a laboratory working with influenza viruses.  Lu et al. [8] point to a possible avian intermediary based of the similarity to some of the isolates from mallard ducks in South Dakota. These alternative theories predict different environments, a swine or avian host, or a laboratory environment, where the unaccounted for ancestors of the H1N1 pandemic virus evolved.

Traditional phylogenetic techniques classify organisms based on genomic similarity. However, due to lack of sampling, it is unlikely that phylogenetic techniques will provide clues about the origins of the pandemic virus, unless new direct ancestors are sequenced. Instead of comparing the genomes, we have compared the evolutionary patterns in different viral species, influenza A and B, and different hosts (avian, human, equine and swine) (Figure 1). In a physics analogy, phylogenetic approaches compare positions and here we compare the direction of the velocities, i.e. the direction of the mutational changes.

**FIGURE 1**

**Figure:**
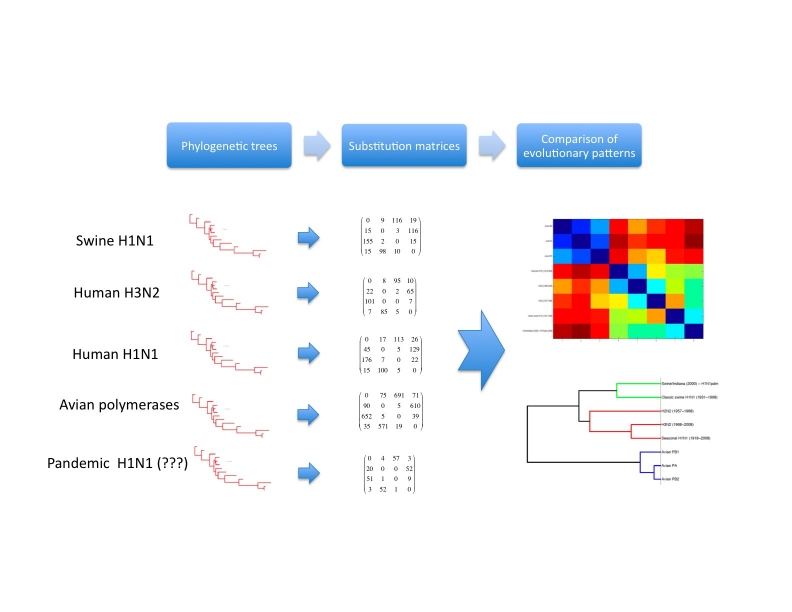

At the end of an evolutionary process, of course, the combination of mutation, selection and chance, is all that is actually observed. The random nature of mutations does not imply that every nucleotide has the same probability of turning into another nucleotide, but rather that there is a distribution of mutations that generate the large amount of possible descendants upon which selection acts. In general, evolutionary changes are hard to observe, as evolution is a slow process, and only an indirect inference of ancestral states is possible. Influenza, however, provides an example of an organism that evolves relatively fast, allowing the inference of properties in evolutionary changes. A previous work [9] pointed to a subtle effect in H3N2 and H1N1 seasonal human influenza A viruses. Viruses that replicate in humans were more likely to change a C into a U in the viral RNA (a G to A change in the mRNA) than vice versa, with the final effect that the number of uracils in human H1N1 seasonal influenza viruses has increased almost 2% since 1918. While the effect is subtle, only a few percent of the substitutions show this bias, its observation was possible because of both the large evolutionary rate of RNA viruses and a historical genomic record since 1918. 

Here we study the full substitution patterns describing the evolution of influenza viruses in different hosts.  We show that equine, avian, human and swine influenza A and B viruses undergo different substitution patterns. Although subtle, the differences between these evolutionary patterns reflect the host where the virus has been replicating. Noticeably, the pattern of substitutions between the swine ancestors of the pandemic virus and one of the earliest examples of the 2009 H1N1 pandemic reveals the same pattern as other swine viruses, implying it evolved in a swine environment during the unobserved part of its evolution

## Results and Conclusions

The genome of influenza has a nucleotide content that depends on the species (A or B viruses), strain and host. A simple measure of evolutionary bias, which is independent of nucleotide content, is the substitution matrix.  For instance, A-G transitions in viral mRNA, the quantities

p(G→A) = (# G to A transitions)/(# Gs)

p(A→G) =(# A to G transitions)/(# As)

can be compared to establish if one direction of transition is preferred over another. The ratio of these quantities represents the probability that a G→A transition would happen versus an A→G transition if the A/G nucleotide content were uniform along the genome.  To be certain that an effect is due to mutational bias, as opposed to some other effect (such as amino acid changes), we can repeat the calculation of these ratios exclusively for synonymous transitions.  In this case, the nucleotide content is replaced by the effective nucleotide content.  For instance, the effective number of G sites in the genomes is the number of Gs that, if they underwent a G→A transition, would cause a synonymous change.

Our study reveals the same bias towards G→A as opposed to A→G transitions in mRNA in all influenza viruses infecting mammals, and a somewhat weaker bias towards C→U vs U→C.  Analysis of 2009 pandemic influenza, by looking at multiple strains in the relatively short period of time since its emergence, shows as expected that it is subject to the same bias as the other influenza viruses.  These results are presented in the table below for synonymous transitions (mRNA sense):

**Table:** 

Subtype	Ancestor	Descendant	p(G→A)/p(A→G)	p(C→U)/p(U→C)
A/H1N1	1918	2008	2.2	1.1
A/H3N2	1976	2008	2.2	1.04
A/H1N1 (2009 pandemic)	-	-	2.3	1.5
A/swine/H1N1	1931	1976	1.9	1.5
A/equine/H3N8	1963	2003	4.3	1.8
B	1940	2008	3.0	1.2

The result is robust to the selection of different isolates from the same strains (for a more detailed study see Supplementary Table ST1).  Non-synonymous substitutions presented the same bias but weaker bias than synonymous substitutions, indicating that the effect is not just the result of amino acid changes and that, rather, is a true bias.  A separate study of the evolutionary bias for the three polymerase segments, which comprise almost half of the genome, does not reveal any distinct pattern for them compared to the other segments, which again indicates that the evolutionary bias is also not associated with a specific set of segments or those segments’ functions.

The biases obtained by observing the evolution of nearly five hundred complete sequences available for 2009 pandemic H1N1 coincide with the bias obtained by observing other mammalian strains over long periods of time. From this, one can conclude that the asymmetric nucleotide substitution pattern is common to all observed mammalian influenza strains and that the short-term evolutionary rate of bias agrees with the long-term rate. The constancy of this evolutionary pattern across mammalian host species and multiple time scales seems to suggest that this bias is due to a general evolutionary mechanism acting on these viruses at multiple time scales.  Whether this bias is caused by an additional mechanism, such as enzymatic activity (a cytidine deaminase, for instance), or immune selection [10], is unclear.

Given the appearance of an asymmetric mutational pattern that exists across mammalian strains, we turn our attention to using this bias in order to establish whether or not the 2009 H1N1 pandemic virus had been evolving in swine prior to its emergence.  We do this by first computing the normalized substitution matrix for each influenza A strain available in avian, human, and swine, using standard maximum likelihood phylogeny.  The substitution matrix reflects the nature of the evolutionary changes, e.g. what is the ratio of transitions vs transversions, mutations in purines vs pyrimidines, etc.  In the absence of any other constraint, the nucleotide content of an organism will evolve to an asymptotic point given by the equilibrium nucleotide composition. This represents the equilibrium frequencies toward which each strain is evolving, which are shown in the table below.

**Table:** 

**Strain**	**A%**	**C%**	**G%**	**U%**
Seasonal H1N1 (1918-2008)	40.2	16.4	16.9	26.5
H2N2 (1957-1968)	40.1	20.3	17.6	22
H3N2 (1968-2008)	39.8	18.4	16.9	24.8
Classic swine H1N1 (1931-1998)	38.1	15.8	21.3	24.7
B	51.3	11.8	16.5	20.3
Equine H3N8	50.3	12.6	14	23.1
Avian PB2	34.2	21.6	17.7	26.5
Avian PB1	34.7	22.3	18.7	24.3
Avian PA	33.5	22.3	18.5	25.7
Swine/Indiana(2000)-H1N1pdm(2009)	40.3	16.8	20.2	22.6

As one can see by simple inspection, there is a clustering amongst influenza A strains by species.  Amongst non-equine influenza A strains, the bias towards greater A nucleotide content seems greatest in human strains, followed by swine, and is somewhat less in avian strains. Figure 2 shows a dendogram of the relation between the different evolutionary processes. It is worth emphasizing that this dendrogram is not a phylogenetic tree, each leave does not represent a particular viral isolate. Each leaf of this dendrogram represents an evolutionary process, for instance, from A/swine/Indiana/2000 (H1N2) to the recent pandemic H1N1 virus. Figure 3 shows graphically the relation between the different strains by their equilibrium frequencies.  In more technical terms, these figures plot the kernel of the substitution matrix describing the most recent common ancestor of the swine polymerases to the current pandemic. Equilibrium frequencies cluster hosts by the evolutionary patterns of the viruses evolving within them, i.e. the human strains cluster together, as do the avian strains.

The equilibrium representing the dynamics of the pandemic from 2000 to its emergence clearly clusters with classical swine H1N1, strongly suggesting that it did indeed evolve in swine in the interval between its last common ancestor and its emergence. This is further demonstrated in the heat map shown in Figure 2, where patterns of nucleotide frequency segregate according to host species, with the hidden swine dynamics clustering in the same pattern as classical swine. In addition to showing a swine specific pattern, the rates are consistent with what one would expect from normal evolution over this period.  One can contrast this with the emergence of the 1977 H1N1 virus, which is believed to have escaped from a lab [11].  In this case the virus showed very few changes from the time it disappeared from the population until it was next detected, implying a literally “frozen” evolutionary pattern.  Here we show that, not only was this virus evolving at a normal rate, but also that it was evolving in a manner consistent with the theory that it was replicating in swine over the unobserved interim. The fact that the ancestors of the H1N1 pandemic strain present similar evolutionary patterns as other swine viruses provides a strong evidence for the ‘unsampled pig herd hypothesis’.

By using host specific patterns imprinted in the genome of a virus, we are therefore able to draw conclusions about its past history. In principle, a similar strategy could be applied in future outbreaks to identify the history of emerging pathogens between the time when they emerged and their last known ancestor, providing valuable information about the environment in which the virus most recently replicated.

**FIGURE 2**

**Figure:**
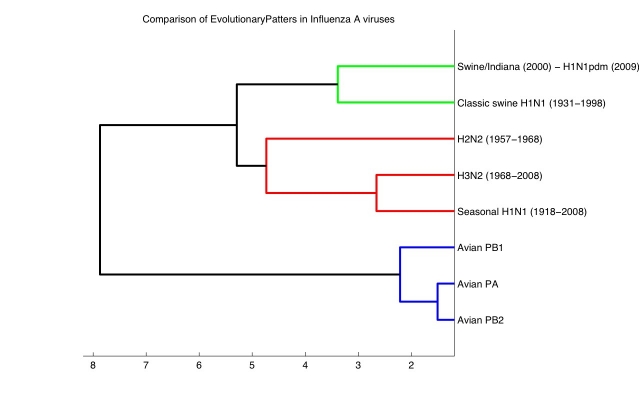

**FIGURE 3**

**Figure:**
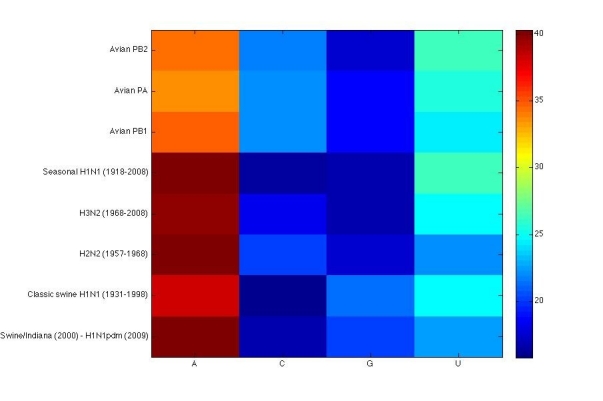

## Materials and Methods

We study the evolution of different influenza virus subtypes by comparing genomes isolated several decades apart in time. This comparison reveals a general mammalian mutational bias across different Influenza A host subtypes to multiple mammalian species and strains. We use the large amount of isolates available and compare all of them simultaneously using phylogenetic analysis. Summing the substitutions in all the branches of the phylogenetic tree we get a larger number of changes for each of the viral subtypes and hosts which enables us to see the difference between them.  To detect mutational bias, we extract synonymous changes and divide them by the number of effective sites, which yields the substitution matrix for synonymous changes. For instance, for the G→A  change the effective number of G sites in the genome is the number of G's that, if they underwent a G→A transition, would cause a synonymous change. In particular

p(G→A) = (# G to A transitions)/(# G's that could go to A synonymously).

These biases were computed for all strains listed in Table 1. 

To examine the host specific effects of this mutational bias, we compute the substitution matrix for synonymous changes at effective sites and normalize the substitution matrix so that all its elements sum to one.  For human and swine strains we calculate this for the three polymerase segments in order to avoid the effects of recombination.   As these rates may be biased by effects such as how far from equilibrium a strain was when it emerged, we compute the kernel of thiis matrix a robust measure of mutational bias.  The kernel of the substitution matrix essentially represents  the equilibrium frequencies of the nucleotides.

We use the 471 available (as of October 3 2009) sequences of 2009 H1N1 pandemic influenza and align them using the A/Mexico/4108/2209 sequence as a reference sequence. Only 403 of them are unique. We use only the coding regions of the eight segments, and for the segments with several reading frames, we choose the longer one (PB1, M1, NS1). Four genomic isolates are missing significant parts of the coding region (A/Luxembourg/43/2009, A/Nonthaburi/102/2009, A/Thailand/CU-H106/2009, A/Thailand/CU-H276/2009) and are removed from the analysis. This distribution reflects the substitution patterns inherent to a particular host, and it does not depend on the nucleotide and amino acid content of the viral genome because of the way it is constructed.

For the seasonal H1N1 virus we use the 41 genomes of the viruses isolated between 1918 and 2008. We build the phylogenetic tree and reconstruct the sequences at the internal nodes   using the Maximum Likelihood method of the PHYLIP package [12]. We use the A/Brevig Mission/1/1918 sequence as an outgroup of the tree. For the HA segment (which is not completely sequenced) we use the A/South Carolina/1/1918 sequence instead. The same technique is used for analysis of different strains. Details of the sequence data used are summarized below.

**Figure:**
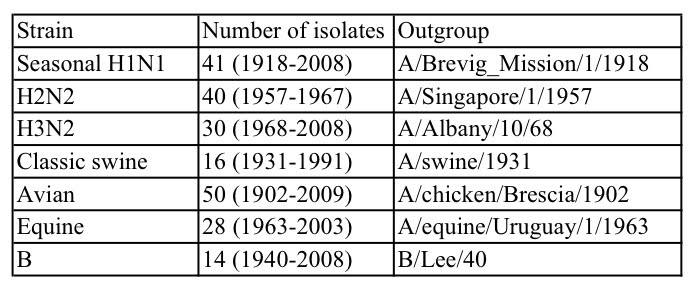

For avian viruses we work with each of the polymerase segments separately since these viruses undergo frequent reassortments.

Hierarchical clustering was performed by considering Euclidean distances with the equilibrium frequencies and linkage by weighted average distance (WPGMA). But the result is identical to other methods of hierarchical clustering (single, average, median, etc) and other choices of distance. To assess the significance of the relation between the evolution of the H1N1 pandemic strain from 2000 (A/swine/H1N2/Indiana/P12439/2000) to 2009 (A/H1N1/Mexico/4108/2009) and the evolution of influenza in different hosts we created a randomized set of substitution matrices and perform a cluster analysis. For each of the hosts and virus subtypes, we randomize the substitution matrix by replacing the actual number of changes observed in the tree corresponding to each of its elements by a Poisson distributed random number with the same mean. We generate 100 replicates for each of the three human seasonal viruses, 100 replicates for each of the three polymerase segments of the avian viruses and 300 replicates for swine viruses. Therefore our bootstrap dataset contains 901 substitution matrices. For each of these matrices we find a set of equilibrium frequencies (assuming that the number of effective sites for each type of change is the same). After that we perform the consensus k-means clustering with these data. The entry which describes the evolution from A/swine/H1N2/Indiana/P12439/2000 to A/H1N1/Mexico/4108/2009 strain gets clustered with 254 other data; 98.8% of the latter being replicates of mammalian evolution matrices, and, in particular, 54.3% H1N1 swine.

## Acknowledgments

B. Greenbaum is the Eric and Wendy Schmidt scholar at the Institute for Advanced Study.  B. Greenbaum and A. Solovyov are joint first authors on the work.

## Funding Information

The work of G. Palacios and W. I. Lipkin was supported by National Institutes of Health awards HL083850 and AI57158 (Northeast Biodefense Center - Lipkin). The work of A. Solovyov has been supported by the National Institutes of Health awards U54 AI57158 (Northeast Biodefense Center - Lipkin) and the Department of Defense. The work of R. Rabadan has been supported by awards from the Northeast Biodefence Center (U54-AI057158-Lipkin) and the National Library of Medicine (1R01LM010140-01).  

## Competing Interests

The authors have declared that no competing interests exist.

## Supplementary Materials

ST1 Mutational bias for different influenza viruses inferred from studying different isolates.

**Figure:**
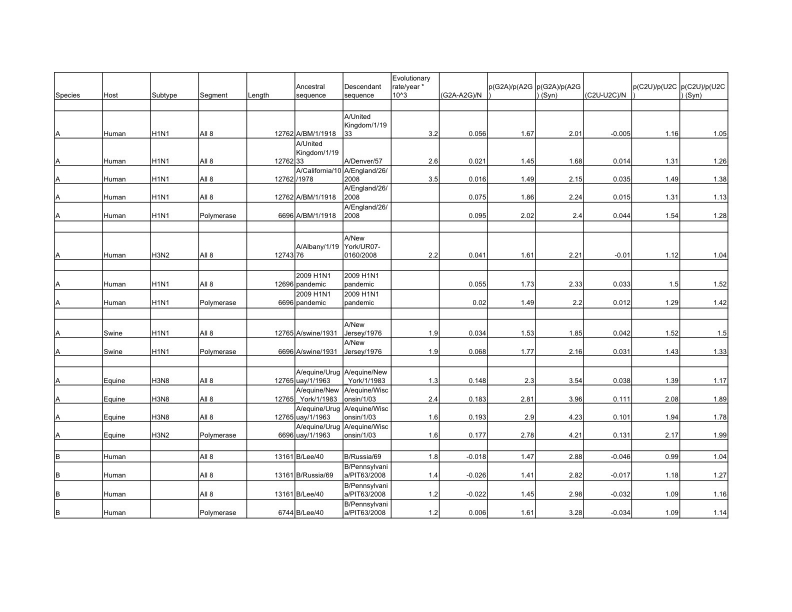

SF1 PCA plot of the data projected onto the first two principal components (a) (b), different colors correspond to the different clusters.

(a)

**Figure:**
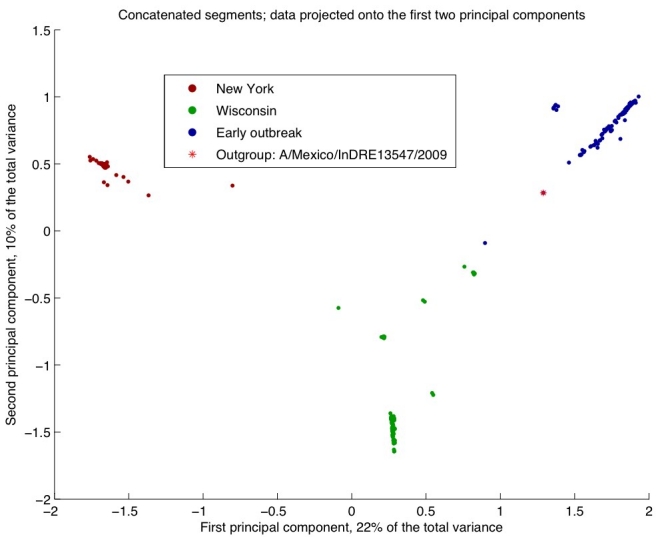

(b)

**Figure:**